# Heat shock protein90 in lobular neoplasia of the breast

**DOI:** 10.1186/1471-2407-8-312

**Published:** 2008-10-28

**Authors:** Flora Zagouri, Afrodite Nonni, Theodoros N Sergentanis, Christos A Papadimitriou, Nikolaos V Michalopoulos, Andreas C Lazaris, Efstratios Patsouris, George C Zografos

**Affiliations:** 1 Breast Unit, 1st Department of Propaedeutic Surgery, Hippokratio Hospital, University of Athens, 108 Vas. Sofias Avenue, 11528, Greece; 2 Associate Professor of Surgery, University of Athens; 101 Vas Sofias Ave, Ampelokipi, Athens 11521, Greece

## Abstract

**Background:**

Heat shock protein 90 (Hsp90) overexpression has been implicated in breast carcinogenesis, with putative prognostic and therapeutic implications. The purpose of this study is to evaluate the immunohistochemical expression of Hsp90 and to examine whether Hsp90 expression is associated with estrogen receptor alpha (ER-alpha) and beta (ER-beta) immunostaining in lobular neoplasia (LN) of the breast.

**Methods:**

Tissue specimens were taken from 44 patients with LN. Immunohistochemical assessment of Hsp90, ER-alpha and ER-beta was performed both in the lesion and the adjacent normal breast ducts and lobules; the latter serving as control. As far as Hsp90 evaluation is concerned: i) the percentage of positive cells, and ii) the intensity was separately analyzed. Additionally, the Allred score was adopted and calculated. Accordingly, Allred score was separately evaluated for ER-alpha and ER-beta. The intensity was treated as an ordinal variable-score (0: negative, low: 1, moderate: 2, high: 3). Statistical analysis followed.

**Results:**

Hsp90 immunoreactivity was mainly cytoplasmic in both the epithelial cells of normal breast (ducts and lobules) and LN. Some epithelial cells of LN also showed nuclear staining, but all the LN foci mainly disclosed a positive cytoplasmic immunoreaction for Hsp90. In addition, rare intralobular inflammatory cells showed a slight immunoreaction. The percentage of Hsp90 positive cells in the LN areas was equal to 67.1 ± 12.2%, whereas the respective percentage in the normal adjacent breast tissue was 69.1 ± 11.6%; the difference was not statistically significant. The intensity score of Hsp90 staining was 1.82 ± 0.72 in LN foci, while in the normal adjacent tissue the intensity score was 2.14 ± 0.64. This difference was statistically significant (p = 0.029, Wilcoxon matched-pairs signed-ranks test). The Hsp90 Allred score was 6.46 ± 1.14 in the LN foci, significantly lower than in the normal adjacent tissue (6.91 ± 0.92, p = 0.049, Wilcoxon matched-pairs signed-ranks test). Within the LN foci, the Hsp90 Allred score was neither associated with ER-alpha, nor with ER-beta percentage.

**Conclusion:**

Hsp90 was lower in LN foci both at the level of intensity and Allred score, a finding contrary to what might have been expected, given that high Hsp90 expression is detected in invasive breast carcinomas. Hsp90 deregulation does not seem to be a major event in LN pathogenesis.

## Background

Heat shock proteins (Hsps) or stress proteins are one of the most evolutionarily conserved classes of molecules to play a fundamental role in the maintenance of cellular homeostasis. Under normal conditions, they act as "molecular chaperones", assisting protein folding, transport and degradation. On the other hand, during stress they prevent aggregation and promote refolding of damaged proteins. Hsps are classified into several families, named according to their approximate molecular weight [1]. Hsp90 is one of the most abundant proteins in mammalian cells [2]. It forms several discrete subcomplexes, each containing distinct groups of co-chaperones that function in these folding pathways.

Elevated Hsp90 expression has been documented in breast cancer [3-5], contributing to the proliferative activity of breast cancer cells. In parallel, Hsp90 overexpression has been interpreted as a means through which breast cancer cells become resistant to various stress stimuli [5]. Interestingly, a recent study has revealed that higher Hsp90 expression may be a marker of poor disease prognosis [6]. It has also been suggested that these family proteins are directly involved in the drug resistance of breast cancer cells [7,8]. As a result of the multifaceted Hsp involvement in breast cancer, pharmacological inhibition of Hsps appears to provide therapeutic opportunities in the field of cancer treatment [9-13]; 17-allylamino, 17-demethoxygeldanamycin (17-AAG), the first Hsp90 inhibitor to undergo clinical development, has yielded promising results [14,15].

However, there is no data reporting on the Hsp expression in response to precancerous breast lesions and lobular neoplasia in particular. According to the most recent WHO classification, lobular neoplasia (LN) includes the designations atypical lobular hyperplasia (ALH) and lobular carcinoma in situ (LCIS) and refers to the entire spectrum of atypical epithelial proliferation originating in the terminal duct-lobular unit, with or without involvement of ducts [16]. Nowadays, it is widely known that LN represents a risk factor and a non-obligatory precursor for the subsequent development of invasive carcinoma in either breast, of either ductal or lobular type [17].

Hsp90 interacts with a complex of proteins which play key roles in breast neoplasia. This complex includes estrogen receptors (ER), tumor suppressor p53 protein, angiogenesis transcription factor HIF-1alpha, antiapoptotic kinase Akt, Raf-1 MAP kinase and a variety of receptor tyrosine kinases, such as erbB2 (reviewed in [14]. Among these proteins, examination of ERs seems an appropriate point to begin with, given the absolute lack of data concerning LN; indeed, in the context of breast carcinogenesis, ERs play a pivotal role (reviewed in [18]). Not being affected by mutations, ERs represent a molecule with particular clinical and therapeutic importance [19].

Concerning ER expression in LN, ER receptor positivity is a well-established feature of lobular carcinoma *in situ *[20,21]. Interestingly, our previous work in LN has demonstrated a significant ER-alpha upregulation and ER-beta downregulation in LN; regarding the ER-alpha/ER-beta ratio, this shift in favor of the numerator may represent a relative proliferative advantage of LN cells. Noticeably though, the two parallel molecular events seemed to exhibit a mutually limiting behavior, according to which greater increase in ER-alpha expression was associated with smaller reduction in ER-beta expression and vice versa [22].

To date, there are no data examining Hsp90 in combination with ER-alpha and ER-beta status in patients whose main lesion is LN. Interestingly, it is tempting to speculate that, given the importance of both ERs and Hsp90, their simultaneous examination may have implications for the relative risk associated with these borderline lesions and suggest opportunities for chemoprevention. To our knowledge, this is the first study to focus on the immunohistochemical expression of Hsp90 and estrogen receptors alpha and beta in LN cases; apart from the immunohistochemical expression of Hsp90 by itself, special attention was paid to the ER-Hsp90 association.

## Methods

Formalin-fixed, paraffin-embedded tissue specimens were taken from 44 patients with LN. The patients' age at operation ranged between 34 and 67 (median age: 48 years). The diagnosis of LN was established by vacuum-assisted breast biopsy or by excisional breast biopsy. Cases of LN coexisting with atypical ductal hyperplasia, ductal carcinoma in situ, invasive lobular carcinoma or invasive ductal carcinoma were excluded.

Hsp90 was immunohistochemically detected using the monoclonal antibody Hsp90 (clone JPB24) (Novocastra supplied by Menarini), while ER-alpha and ER-beta receptors were immunohistochemically detected with the commercially available ER6f-11 and ER-beta (clone EMRO2) (Novocastra supplied by Menarini) antibodies. All were visualized using an avidin-biotin detection system. Antigen retrieval was achieved in 0.001 M citrate buffer (pH = 6.0) at 85°C for 18 h. Immunohistochemical assessment of Hsp90, ER-alpha and ER-beta was performed both in the lesion and the adjacent normal breast ducts and lobules; the latter serving as control. Negative controls were assessed by omitting the primary antibody.

As far as Hsp90 evaluation is concerned: i) the percentage of positive cells, and ii) the intensity was separately analyzed. Additionally, the Allred score was adopted and calculated; due to the lack of scoring system taking simultaneously into account both intensity and percentage of Hsp90 staining.

Accordingly, Allred score was separately evaluated for ER-alpha and ER-beta. In addition, to assess the clinical relevance of ER-alpha and ER-beta expression, cases were designated as negative (0%), equivocal (less than 10%) and positive (≥ 10%), as presented elsewhere [23,24].

In all cases, the area of maximum staining intensity was preselected on each slide and a minimum of 100 cells were evaluated in the designated area. The immunohistochemical evaluation was performed independently by two consultant histopathologists (AN and AL).

The intensity was treated as an ordinal variable-score (0: negative, low: 1, moderate: 2, high: 3). Statistical analysis was performed with STATA 8.0 statistical software (Stata Corporation, College Station, TX, USA). Due to deviation from the normal distribution, non-parametric statistics were chosen. The statistic performed in each case is mentioned in parentheses in the text.

Informed consent was obtained by all participants in this study. This study has been approved by the local Ethics Committee, in compliance to the Helsinki Declaration.

## Results

**Figure 1:**
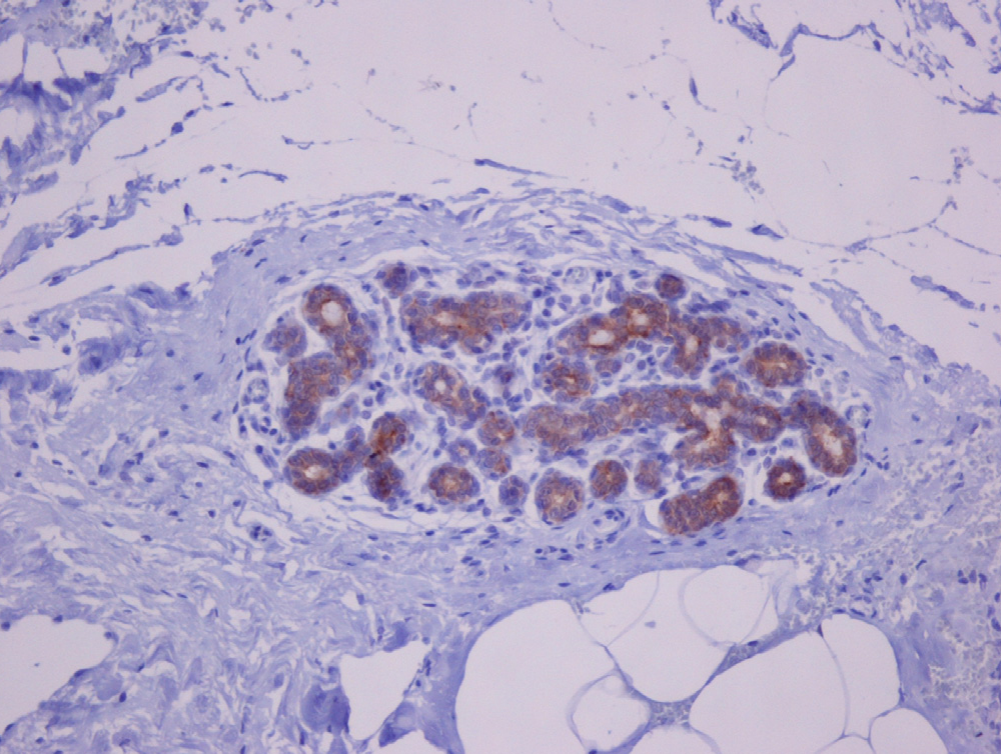
**Strong cytoplasmic HSP90 immunoreactivity in the epithelial cells of lobule with slight dilation of the acini.** Rare inflammatory cells are slightly immunoreacted, as well (×100).

**Figure 2:**
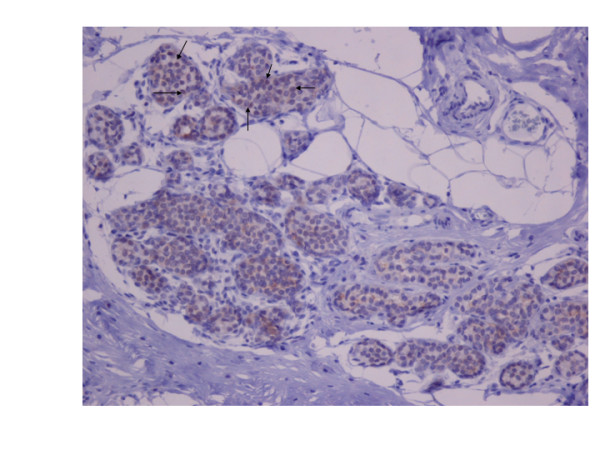
**Moderate HSP90 cytoplasmic immunoreaction in the epithelial cells of LN.** Note the nuclear imunoreaction of some epithelial cells in the upper half of the figure (×200).

**Figure 3:**
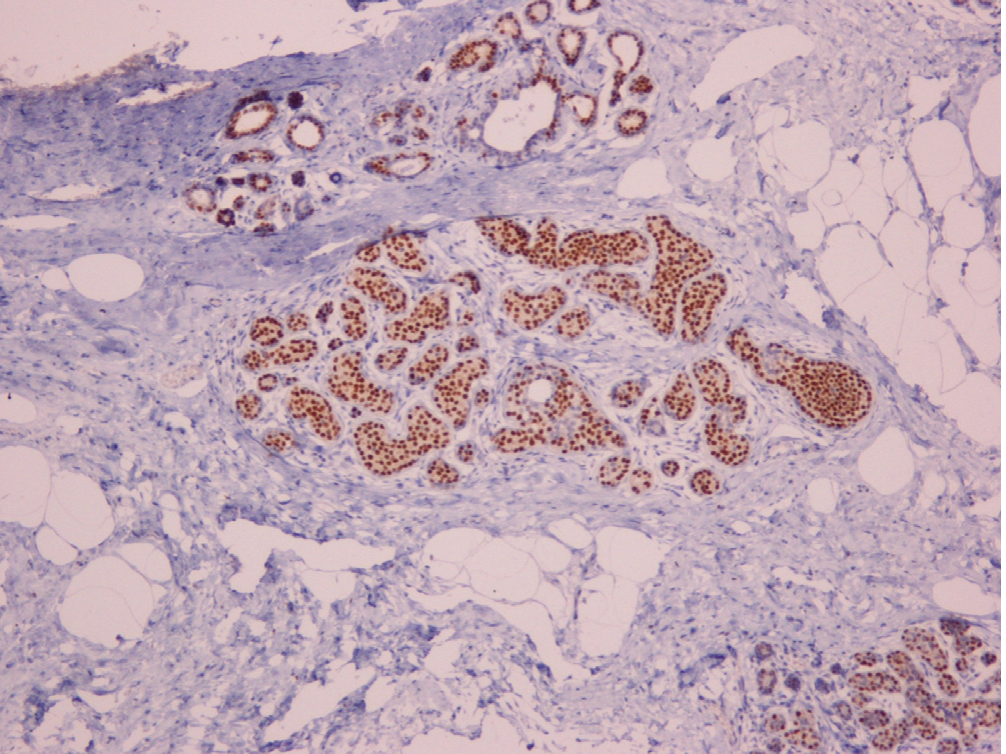
Immunohistochemical nuclear expression of ER-alpha in LN lesions (×100).

**Figure 4:**
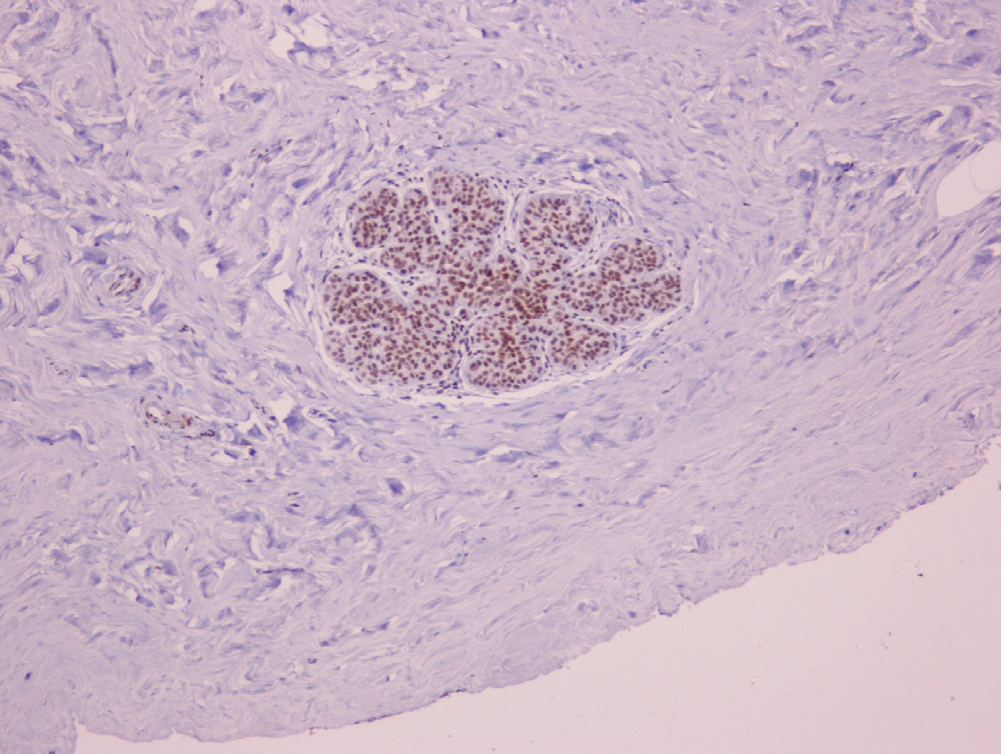
Immunohistochemical nuclear expression of ER-beta in LN lesions (×100).

Hsp90 immunoreactivity was mainly cytoplasmic in both the epithelial cells of normal breast (ducts and lobules) (figure 1) and LN (figure 2). Some epithelial cells of LN also showed nuclear staining (figure 2), but all the LN foci mainly disclosed a positive cytoplasmic immunoreaction for Hsp90; the percentage of these positive cells and the staining intensity were evaluated. In addition, rare intralobular inflammatory cells showed a slight immunoreaction (figure 1). As far as ER is concerned, moderate to strong nuclear ER-alpha (figure 3) and ER-beta (figure 4) immunoreactivity was detected in epithelial cells of normal ducts and lobules and in LN. Additionally, ER immunostaining has been observed on some lymphocytes.

The percentage of Hsp90 positive cells in the LN areas was equal to 67.1 ± 12.2%, whereas the respective percentage in the normal adjacent breast tissue was 69.1 ± 11.6%; the difference was not statistically significant (p = 0.679, Wilcoxon matched-pairs signed-ranks test).

**Figure 5:**
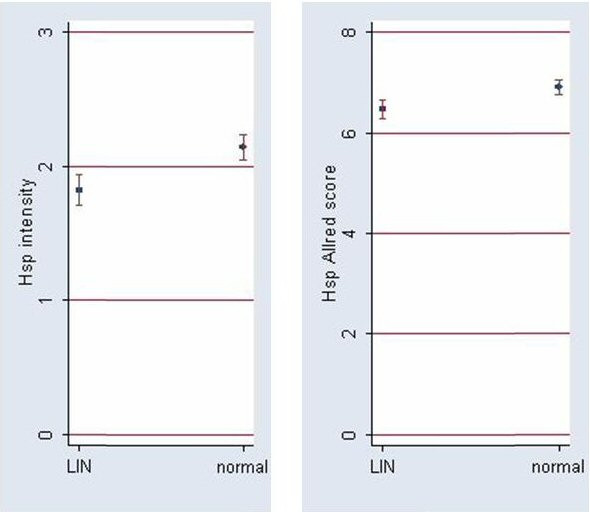
**Hsp90 intensity and Allred scores (mean ± SE) in LN lesions and normal adjacent breast tissue.** Both scores are significantly lower in LN lesions (cf. Results).

The intensity score of Hsp90 staining was 1.82 ± 0.72 in LN foci, while in the normal adjacent tissue the intensity score was 2.14 ± 0.64. This difference was statistically significant (p = 0.029, Wilcoxon matched-pairs signed-ranks test). The Hsp90 Allred score was 6.46 ± 1.14 in the LN foci, significantly lower than in the normal adjacent tissue (6.91 ± 0.92, p = 0.049, Wilcoxon matched-pairs signed-ranks test). The two statistically significant findings are depicted in Figure 5.

Concerning ERs, it should be stressed that all LN cases were above the 10% positivity threshold both regarding ER-alpha and ER-beta; as a result, all LN cases were designated as alpha positive/beta positive. The more elaborate analysis by means of the Allred score was in line with the results previously reported [22]; in LN the Allred score for ER-alpha was higher than in the adjacent normal breast ducts and lobules (6.78 ± 1.19 vs. 6.33 ± 1.28; p = 0.047, Wilcoxon matched-pairs signed-ranks test), but lower as far as ER-beta was concerned (6.45 ± 1.18 vs. 7.22 ± 0.69; p < 0.001, Wilcoxon matched-pairs signed-ranks test)

Within the LN foci, the Hsp90 Allred score was neither associated with ER-alpha (Spearman's rho = -0.039, p = 0.823), nor with ER-beta percentage (Spearman's rho = -0.027, p = 0.878). Similarly, with regard to percentage and intensity no statistically significant associations were documented between Hsp90 and ER-alpha, ER-beta. Likewise, the Hsp90 Allred score was neither associated with ER-alpha (Spearman's rho = -0.178, p = 0.267), nor with ER-beta percentage (Spearman's rho = -0.012, p = 0.943) within the normal adjacent tissue. Similarly, no statistically significant associations existed between the constituents.

In an attempt to evaluate mutually limiting phenomena (cf. [22]), the difference in Allred score for Hsp90 [Allred score in LN - Allred score in the normal adjacent tissue] was comparatively assessed with the respective differences for ER-alpha and ER-beta. However, no statistically significant associations existed (Spearman's rho = 0.100, p = 0.561 for ER-alpha, and Spearman's rho = -0.054, p = 0.757 for ER-beta).

## Discussion

In comparison to the adjacent normal breast lobules and ducts, Hsp90 expression was lower in LN foci both at the level of intensity and Allred score. This finding seems contrary to what might have been expected, as it is widely accepted that LN represents a precursor lesion and that Hsp90 overexpression is detected in invasive breast carcinomas [4,6]. For the optimal interpretation of our results, it should be kept in mind that LN is a non-obligatory precursor of breast cancer. In other words, the molecular events therein may be self-limiting and may not necessarily reflect those more extensive in invasive breast cancer. In addition, LN lesions are benign lesions containing cells not yet subject to the stresses occurring in invasive breast cancer and thus a stress response seems not to have been triggered at that neoplastic phase. On the other hand, the present findings may indicate that the relative proliferation advantage in LN cells is an Hsp90-independent process.

Noticeably, Hsp90 levels (as reflected both upon the percentage and staining intensity) were not associated with ER-alpha and ER-beta status. This seems intriguing, as Hsp90 is closely associated with the ER receptor signaling pathway, playing an essential role in the stability and function of steroid hormone receptors [25]. Indeed, *in vitro *evidence in the literature actively implicates Hsp90 in the regulation of ER expression. More specifically, it has been suggested that Hsp90 protects ER-beta against proteasomal degradation [26]; accordingly, Hsp90 inhibitors have been shown to result in proteasome-mediated degradation of ERs (reviewed in [14]).

Worthy of note, in the present study the above ER-Hsp90 association data are not confirmed at the level of immunohistochemical expression in LN. Interestingly, the lack of ER-Hsp90 association pertained to both LN and adjacent normal breast tissue. These findings may indicate that LN represents a condition maintaining regulatory mechanisms present in the normal status, thereby suggesting that ER up-regulation and expression variability seem not to be associated with Hsp90 perturbations at the context of LN. Nevertheless, the inability to document the ER-Hsp90 association may be partly due to the method performed (i.e. immunohistochemistry); quantitative methods might be more sensitive regarding this association.

To date, studies have examined Hsp90 status in breast cancer [4-6,8] without specifically focusing on lobular lesions and carcinomas. Thus, it remains unclear whether the herein documented results are indicative *per se *of the status in lobular carcinogenesis or in precursor lesions in general. Undoubtedly, further studies exclusively based on invasive lobular carcinomas are essential to extrapolate the present findings onto the continuum of lobular cancer pathogenesis. Whether ER- Hsp90 independence disappears in invasive lobular carcinoma, remains yet to be clarified.

The exclusive examination of LN lesions is a distinct advantage of this study. However, it was not without its technical limitations. More specifically, as mentioned above, the lack of an automated quantitative procedure may have clouded some associations. Notwithstanding, Hsp90 downregulation is not contestable given its clear expression in a relatively less sensitive assessment. To ensure the objectivity of the assessment, the percentage and intensity were assigned by two independent pathologists blind to one another's results.

In our cases, some epithelial cells of LN foci showed nuclear Hsp90 localization. This finding has already been reported in invasive breast carcinomas and has been correlated with MHC class I expression [27].

## Conclusion

In conclusion, it can be said that Hsp90 expression was lower in LN foci both at the level of intensity and Allred score, a finding contrary to what might have been expected, given that high Hsp90 expression is detected in invasive breast carcinomas. Hsp90 deregulation does not seem to be a major event in LN pathogenesis. As regards LN, Hsp90 does not seem to be associated with estrogen receptor status. Further studies adopting quantitative procedures and assessing mRNA levels are needed. This study may prompt other studies evaluating Hsp90 expression in invasive lobular carcinoma as well as other precursor lesions.

## Abbreviations

Hsp: Heat shock protein; ER-alpha: estrogen receptors alpha; ER-beta: estrogen receptors beta; LN: lobular neoplasia; ALH: atypical lobular hyperplasia; LCIS: lobular carcinoma in situ.

## Competing interests

The authors declare that they have no competing interests.

## Authors' contributions

FZ: conceived the idea, participated in the design of the study and assisted in the writing of the manuscript. AN: performed the pathological evaluation. TS: participated in the design of the study, performed the statistical analysis and assisted in the writing of the manuscript. CP: participated in the design of the study and evaluated critically the manuscript. NM: performed vacuum-assisted breast biopsy and open surgery. AL: performed the pathological evaluation. EP: participated in the design of the study and evaluated critically the manuscript. GZ: conceived of the study, participated in its design, performed vacuum-assisted breast biopsy and open surgery and evaluated critically the manuscript.

## Pre-publication history

The pre-publication history for this paper can be accessed here:


